# Perspective: Leveraging Electronic Health Record Data Within Food Is Medicine Program Evaluation: Considerations and Potential Paths Forward

**DOI:** 10.1016/j.advnut.2024.100192

**Published:** 2024-02-23

**Authors:** Christopher R Long, Amy L Yaroch, Carmen Byker Shanks, Eliza Short, Elise Mitchell, Sarah A Stotz, Hilary K Seligman

**Affiliations:** 1 Gretchen Swanson Center for Nutrition, Omaha, NE, USA; 2 Department of Food Science and Human Nutrition, Colorado State University, Fort Collins, CO, USA; 3 Division of General Internal Medicine, University of California San Francisco, San Francisco, CA, USA

**Keywords:** Food is Medicine, electronic health records, food insecurity, program evaluation, health care utilization, produce prescriptions

## Abstract

Government, health care systems and payers, philanthropic entities, advocacy groups, nonprofit organizations, community groups, and for-profit companies are presently making the case for Food is Medicine (FIM) nutrition programs to become reimbursable within health care services. FIM researchers are working urgently to build evidence for FIM programs’ cost-effectiveness by showing improvements in health outcomes and health care utilization. However, primary collection of this data is costly, difficult to implement, and burdensome to participants. Electronic health records (EHRs) offer a promising alternative to primary data collection because they provide already-collected information from existing clinical care. A few FIM studies have leveraged EHRs to demonstrate positive impacts on biomarkers or health care utilization, but many FIM studies run into insurmountable difficulties in their attempts to use EHRs. The authors of this commentary serve as evaluators and/or technical assistance providers with the United States Department of Agriculture’s Gus Schumacher Nutrition Incentive Program National Training, Technical Assistance, Evaluation, and Information Center. They work closely with over 100 Gus Schumacher Nutrition Incentive Program Produce Prescription FIM projects, which, as of 2023, span 34 US states and territories. In this commentary, we describe recurring challenges related to using EHRs in FIM evaluation, particularly in relation to biomarkers and health care utilization. We also outline potential opportunities and reasonable expectations for what can be learned from EHR data and describe other (non-EHR) data sources to consider for evaluation of long-term health outcomes and health care utilization. Large integrated health systems may be best positioned to use their own data to examine outcomes of interest to the broader field.

**Statement of Significance**

This Perspective article describes recurring challenges related to using electronic health records (EHRs) in Food is Medicine evaluation, particularly in relation to biomarkers and health care utilization. This article also outlines potential opportunities and reasonable expectations for what can be learned from EHR data and describes other (non-EHR) data sources to consider for evaluation of long-term health outcomes and health care utilization.

Food is Medicine (FIM) programs are food-based programs that address both food and nutrition security in the prevention or management of diet-related health conditions [1,2]. Although organized in various ways, FIM programs often involve partnerships among health care organizations, community-based organizations, and other supporting organizations (eg, food retailers, food producers, and program evaluators). These partners often share the goal to improve food and nutrition security, diet quality, health, and health care utilization among people experiencing food and nutrition insecurity and chronic disease.

In 2022, the Biden-Harris Administration hosted the White House Conference on Hunger, Nutrition, and Health, coinciding with the release of a White House strategy calling for expanded insurance coverage of FIM programs across the United States [3]. At the time of writing, 10 US states have active or pending Medicaid waivers to deploy experimental, pilot, or demonstration projects to address food insecurity when medically appropriate [4]. Alongside these governmental efforts, health care systems and payers, philanthropic entities, advocacy groups, nonprofit organizations, for-profit companies, and researchers are making the case for FIM programs to become reimbursable within health care services [1,[5], [6], [7], [8]].

FIM researchers are working urgently to build evidence for FIM programs’ cost-effectiveness by showing improvements in health outcomes and health care utilization. However, primary collection of this data is costly, difficult to implement, and burdensome to participants. Electronic health records (EHRs) offer a promising alternative to primary data collection because they provide already-collected information from existing clinical care. A few FIM studies have leveraged EHRs to demonstrate positive impacts on biomarkers or health care utilization (eg, HbA1c, blood pressure, clinic visits, medication use, and hospitalizations) [[9], [10], [11], [12], [13]]. However, using EHRs for these purposes poses numerous challenges.

A prominent example of FIM programs is the United States Department of Agriculture’s (USDA) Gus Schumacher Nutrition Incentive Program (GusNIP) Produce Prescription (PPR) Program. GusNIP PPR projects engage health care partners to prescribe free or reduced-cost fresh fruits and vegetables with the goals of increasing access to and consumption of fruits and vegetables, reducing food insecurity, and improving health care utilization and associated costs [14]. The authors of this commentary serve as evaluators and/or technical assistance providers with the USDA’s GusNIP National Training, Technical Assistance, Evaluation, and Information Center (NTAE). We work closely with over 100 GusNIP PPR projects, which, as of 2023, span 34 US states and territories. Several of the authors also have experience implementing FIM programs.

Many GusNIP PPR projects initially plan to use EHR data in their evaluations but encounter insurmountable challenges [8]. To better support them, we have consulted over the previous 4 years with PPR and FIM implementers, health informatics experts, health systems’ EHR managers, and FIM researchers with experience using EHRs to evaluate programs. In this commentary, we describe recurring challenges related to the use of EHRs in FIM evaluation, particularly in relation to biomarkers and health care utilization. We also outline potential opportunities and reasonable expectations for what can be learned from EHR data and provide other data sources (ie, non-EHR) to consider for evaluation of long-term health outcomes and health care utilization.

It is important to note that the challenges and opportunities for using EHRs in evaluation are broader than FIM. The challenges do not originate from any flaw in FIM partnerships. Evaluators of initiatives in other focus areas, such as quality improvement [[15], [16], [17], [18]], palliative care [19], cardiovascular prevention services [[20], [21], [22], [23], [24], [25]], hypertension control [26], and informatics [27], have described some of the same challenges and opportunities. Although we write from the context of FIM, the breadth of these challenges and opportunities leads us to include some general recommendations that no FIM partnership can address on its own.

## What Challenges Do FIM Programs Face in Using EHR Data?

In 2022, 86% of PPR projects funded by GusNIP reported intention to use EHR data. By the end of their funding cycles, almost none had been successful—despite support from the NTAE. What happened in the interim? In general, health care partners are committed supporters of the project at the time the grant application is being prepared and frequently indicate a desire and willingness to share EHR data. However, challenges emerge when processes for EHR access, sharing, and analysis begin. Table 1 summarizes potential challenges related to data access and sharing and some potential paths forward for FIM partnerships. For programs where EHR data must be shared outside of the health care organization (eg, to an external evaluator or community-based partner), a common challenge is an inability to mitigate the data privacy and liability concerns of the health care partners. Health care organizations are reluctant to risk reputational and financial liability that could arise from breaches of patient privacy from data shared with other organizations in the FIM partnership. From the perspective of the health care organization, the benefits of sharing EHR data with other partners may not outweigh the risks of unintentional or malicious privacy breaches. Many discussions about EHR data sharing end at this point of concern.

**TABLE 1:** Potential challenges and opportunities related to EHR data access and sharing for FIM evaluation

Potential challenges related to accessing and sharing EHR data for FIM evaluation
Health care partner unwilling/unable to share EHR data outside of organization due to privacy and liability concerns
Health care partner and other partners have difficulty reaching agreement on BAA or DUA
Insufficient staffing expertise to extract and transfer EHR data
Limited budget to support staff to conduct the work to extract and transfer EHR data
Inadequate expertise and/or security for external partner to receive, store, or analyze EHR
Potential opportunities for augmenting usefulness and feasibility of EHR data for FIM evaluation
Hold early candid discussions (eg, prior to seeking funding) among partners to identify and manage anticipated challenges to data sharing, including budget, staffing, and organizational policies
Assign EHR data analysis to health care partner staff, who share results—but not data—externally
Measure FIM impact using patient-reported outcomes, which could be measured using survey outside of EHR system
Measure FIM health care utilization using self-reported measures of utilization, which could be measured using survey outside of EHR system

BAA, business associate agreement; DUA, data use agreement; EHR, electronic health record; FIM, Food is Medicine.

The next step in the data sharing process is negotiating a data sharing agreement acceptable to all partners. Health care organizations often propose that FIM partners enter into a business associate agreement (BAA), under which partners receiving the data assume potential financial liabilities of a privacy breach. Instead of a BAA, the non–health care FIM partners may prefer to establish a data use agreement (DUA). Similar to a BAA, the DUA requires FIM partners to safeguard data they receive, but a typical DUA does not implicate the same level of potential liability as a BAA. For this reason, the level of detail in data shared by health care organizations under a DUA may be less than that shared under a BAA. For example, under a DUA, health care partners are frequently only willing to share aggregated and deidentified data, which may not allow for the rigorous detailed evaluation or analyses originally planned.

Negotiating DUAs often requires substantial time investment. We tracked DUA-related negotiations among a subset of PPR grantees and found that negotiations often required dozens of email exchanges among grantee partners, lawyers, and evaluators. One partnership exchanged over 100 emails. In some cases, finalized negotiations also needed to be reopened when it was discovered that data necessary for the analysis were not covered in the original DUA.

The outcomes of these negotiations can reflect significant power imbalances due to differential access to expertise in health care data regulations (eg, when one partner has an in-house legal team with expertise in health care data and another partner struggles to afford fee-for-service legal representation). For each partner involved in the negotiations (eg, when the partnership includes a health care organization, a community-based organization, and an external evaluator), complexity increases. Throughout the negotiations, each partner must weigh the burdens and liabilities assumed by each organization relative to data sharing’s potential benefit to the partnership.

Extraction and transfer of EHR data create additional challenges. Many grant applications fail to budget for the substantial staff time required for health care partners to query, extract, prepare, and share EHR data with other partners. Some health care organizations—particularly small ones, such as clinic sites—may not have any staff trained to perform these functions. Staff from large and small health care organizations warned of weeks-long or months-long queues to query, prepare, or analyze data.

On the receiving end, staff from organizations outside health care does not always have the training, data management plans, and technological solutions to ensure the data are transferred and stored in a way that minimizes risk of privacy breaches. Data sharing is even more complex when programs engage multiple health care organizations. Differences in their EHR systems and internal policies may require that programs use different procedures for extracting and transferring data with each health care partner.

Once EHR data reach the analyst, a data management expert must prepare the data set for analysis—a step that often uncovers numerous additional challenges. For example, data fields expected to be used in the analysis may not contain meaningful and relevant information in a usable format. Complex medical information may need to be recoded into a smaller number of simple categories (eg, Veldheer et al. [12] created a binary yes/no variable from EHR data to represent patients’ “changes in diabetes medications,” which required integrating information from data fields representing starting, stopping, removing, adding medications, or increasing/decreasing medication dosages). A new query, extraction, or data transfer may be needed to address omissions or misspecifications in the initial data transfer.

For FIM partnerships with multiple health care organizations, data fields from different organizations must be harmonized into single variables (even height, weight, and blood pressure are recorded differently in different systems, not to mention more qualitative fields such as program referrals). Effort required for data harmonization increases dramatically with the addition of each new EHR. Even EHR systems provided by the same software vendor (eg, EPIC) vary from organization to organization; a single vendor may offer a range of software packages with diverse versions, specifications, and options that impact data integration. As experts frequently report, “If you’ve seen one EHR, you’ve seen one EHR.” Each expert with whom we consulted expressed concern about the effort required to aggregate data from multiple EHR systems.

## What Can FIM Programs and Evaluators Expect From Analyzing EHR Data?

EHR systems include many types of data of interest for FIM programs and evaluators: demographics, medical and social histories, diagnoses, prescribed medications, laboratory values, and utilization of services. EHRs may include results of screening for food and nutrition insecurity or receipt of FIM services. However, the potential usefulness of EHR data is balanced against EHR data content limitations. Table 2 summarizes potential challenges related to EHR data content and analysis and some potential paths forward for FIM partnerships.

**TABLE 2:** Potential challenges and opportunities related to EHR data content and analysis for FIM evaluation

Potential challenges related to EHR data content and analysis for FIM evaluation
EHR does not include much if any data from the times immediately prior to enrollment and/or during the follow-up period
Population(s) of interest visit multiple health care providers and/or lacks regular access to care
EHR data include too broad a range of utilization, not all of which is relevant to the health conditions addressed by the FIM program and which may include utilization that is “appropriate or inappropriate, of high or low quality, and of high or low cost” [28]
EHR does not include FIM program dosage/utilization
Potential opportunities to augment EHR data content and analysis for FIM evaluation
Augment extracted EHR data with participant surveys to ensure evaluators can assess program impact on variables of interest and time periods of interest, which may not be reflected in data in every participant’s EHR
Focus utilization analyses on a specific kind of utilization (eg, preventive care visits)
Ensure that analysis plan differentiates between utilization intended to decrease (eg, emergency department visits) vs. utilization the intervention intended to increase (eg, preventive care visits)
Begin planning early to link EHR data with data that captures the FIM services each patient received data (eg, food vouchers received and redeemed)

EHR, electronic health record; FIM, Food is Medicine.

Many FIM evaluations aim to use a longitudinal study design to track changes in patients’ biomarkers or health care utilization over time. These designs usually require a comparison of values collected at or near the time of program enrollment to data collected at or near the time of program completion. But, many FIM participants do not interact with the health care organization during these periods. There may not be any useful EHR data from patients who have no encounters with the health care organization during the months immediately prior to FIM program enrollment or the months surrounding FIM program completion. For example, if a patient with type 2 diabetes who received 3 months of food vouchers does not return to the clinic for 9 months after program completion, there will not be a relevant postparticipation HbA1c measurement in that patient’s EHR.

To address these gaps in the data, many analyses allow baseline and follow-up data from broad periods that may not reflect program participation; for example, some patients’ first “follow-up HbA1c” value may not be collected and recorded in the EHR until 6 months after they completed the program. Broader periods increase the number of participants by including those who less frequently encounter the health care organization, and the larger analytic sample results could be interpreted as an improvement simply because a larger sample usually implies more statistical power. However, these values are likely to bias estimates of effectiveness toward the null (or lessen the apparent effectiveness of the programs). For example, baseline EHR biomarkers collected after a patient receives program services may not reflect a true baseline and exposure to the program prior to collection of the biomarker reduces the amount of improvement that can be shown for that patient when baseline and follow-up biomarkers are compared. Similarly, follow-up EHR biomarkers collected several months after a patient’s program participation has ended may no longer reflect the full effects of the program, reducing the amount of improvement that can be shown for that patient when baseline and follow-up biomarkers are compared. The tradeoff between increased sample size and increased relevance of EHR data is difficult to avoid.

Numerous other complexities complicate data analysis. EHRs usually do not include services patients receive from other health care systems (eg, other clinics, urgent care providers, and emergency departments). Because many FIM programs are intended to reach people with vulnerabilities to limited health care, this concern is particularly pertinent to FIM evaluation. For patients who regularly receive care from the FIM partnerships’ health care organizations, EHRs may include a wide range of care, not all of which is relevant to the health conditions addressed by the FIM program [28].

Reducing health care utilization is one mechanism by which FIM programs are often described as reducing health care costs. However, some FIM programs are designed to increase specific types of health care utilization in the short-term (with the goal of reducing intensive and costly health care utilization further into the future). For example, if a patient with type 2 diabetes enrolled in a FIM program is more likely to receive preventive diabetes services, this could reduce costly health care utilization in the long-term, even while health care utilization increases over the period of the study [13,29]. Analyses must, therefore, take on the difficult task of differentiating between utilization patterns that are likely to increase costs in the short-term and reduce costs in the long-term and those that are likely to increase costs in both the short-term and long-term.

Moreover, EHRs do not typically include detailed data about FIM services (eg, number and dollar amount of monthly fruit and vegetable vouchers redeemed). These records are important to evaluators but are most often held by FIM partner organizations outside the health care organization. Thus, to incorporate any data about engagement with FIM services, patient identifiers in the EHR must be matched to service records without violating DUAs or BAAs among the FIM partners. Early planning across organizations is necessary to ensure FIM service data are recorded in a manner that allows accurate matching within the organizations’ available staff time and budgets (eg, in shareable file formats that include shareable identifiers).

## What Non-EHR Data Sources Can Be Used to Evaluate Biomarkers and Health Care Utilization?

The above-described challenges incentivize many FIM partners to explore alternatives. Table 3 summarizes potential challenges and opportunities related to non-EHR data sources and some potential paths forward for FIM partnerships.

**TABLE 3:** Potential challenges and opportunities related to non-EHR data (biomarkers, claims, and self-report data) for FIM evaluation

Potential challenges related to biomarkers, claims, and self-report data for FIM evaluation
Biomarker data are expensive to collect and use (eg, to cover staff time, supplies, and analysis)
Claims data are difficult to access and use
Claims data include only insured people
Self-report data are subject to bias
Potential opportunities to leverage biomarkers, claims, and self-report data for FIM evaluation
Seek funding to support biomarker data collection and/or access to payer data or state claims databases
Align evaluation questions with data sources that are accessible to a particular program (eg, FIM program participation records can be used to evaluate the effectiveness of participant retention strategies)
Plan early to allow linking participant-level data across multiple data sources (eg, linking FIM program participation records with biomarkers, which can be used to evaluate the effects of FIM program dosage received on health indicators)
If designing a self-report survey about health care utilization or FIM program impact, include existing widely used and validated survey items wherever possible rather than developing new (untested) items

EHR, electronic health record; FIM, Food is Medicine.

For example, some FIM partners collect their own biomarker data by inviting participants to attend data collection appointments timed to coincide with optimal baseline and follow-up periods. However, this strategy poses financial and staffing challenges. It is impermissible to bill participants’ insurance for laboratories used solely for program evaluation and not as part of regular clinical care. Staff time for coordinating appointments and for data collection, compensation for participants’ time, and data collection equipment and supplies are expensive. Staff must also be specially trained to collect many biomarkers, particularly laboratory values.

For health care utilization data, claims data can offer a more comprehensive account of health care services received than can EHR data. Claims data often include diagnosis codes, procedures, and treatments received and costs, so can be valuable tools for evaluation. At the time of writing this commentary, large-scale evaluations using claims data are underway to study health care utilization of FIM participants covered by specific insurers (eg, Elevance Health [30], Kaiser Permanente [31], and Veterans Health Affairs [32]).

However, claims data are not a feasible solution for most FIM partnerships. Access to these data is difficult and often requires the participation of a health care payer and/or the capacity, expertise, and funding required to access state databases. For FIM partnerships able to access claims data, the data will include only participants covered by a particular health insurance entity whose claims are included in the database from which the data are drawn. Because many FIM programs target recruitment toward people with low incomes who may be particularly likely to lack health insurance coverage or use multiple sources of coverage, claims data may exclude a considerable proportion of FIM participants. Asking participants directly about their health care utilization using structured surveys can mitigate some of these challenges but comes with its own limitations and challenges (eg, cost, biases in recollection and reporting).

## Conclusions

There is significant enthusiasm now for identifying ways that FIM programs can become reimbursable health care services. This enthusiasm has contributed to a sense of urgency to demonstrate positive health impacts and cost-effectiveness. EHRs are regularly promoted as a means to demonstrate these outcomes. However, the use of EHRs in evaluation is challenging for many FIM partnerships.

Extraction and transfer of EHR data for use in program evaluation are complex and time intensive. FIM partners engaging in these activities require resources to query, extract, prepare, and share EHR data with partners. Those resources have not yet been widely deployed. There are also many complex decisions related to defining the timing and types of health care services and biomarkers to be used in analyses to demonstrate impacts and cost-effectiveness of FIM programs. For example, it is not always clear which types of biomarkers and health care utilization should be expected to improve by participation in a specific FIM program for a specific amount of time in a specific population of participants. Many FIM partnerships lack specialized expertise to develop analytic plans for complex data.

We encourage FIM partners to consider these challenges before committing to using EHR data in applications to their funders. TABLE 1, TABLE 2, TABLE 3 present approaches FIM partners can use to evaluate the impact of their programs, whether (or not) they have the resources and expertise required to leverage EHR data. The GusNIP NTAE’s Nutrition Incentive Hub website (nutritionincentivehub.org) provides further resources to support FIM evaluation and contact information to access technical support. For now, large integrated health systems may be best positioned to use their own data to examine outcomes of interest to the broader field, rather than relying on the sharing of data with partners for evaluation. To investigate FIM programs’ impact on health outcomes and health care utilization, we ask funding agencies to fully support the expenses associated with EHR and claims data access, extraction, and analysis. To investigate broader impact of FIM programs, we also call for funding agencies to fully support the expenses associated with gathering biomarkers and self-reports from participants over time and comprehensive approaches to evaluate FIM’s effects on communities and food systems.

## Author contributions

The authors’ responsibilities were as follows – CRL, ALY, CBS, ES, EM, SAS, and HKS wrote the article; CRL: had primary responsibility for final content; and all authors: read and approved the final manuscript.

### Conflict of interest

The authors declare that they have no known competing financial interests or personalrelationships that could have appeared to influence the work reported in this paper.

### Funding

The Nutrition Incentive Program Training, Technical Assistance, Evaluation, and Information Center (NTAE) is supported by Gus Schumacher Nutrition Incentive Program grant no. 2019-70030-30415/project accession no. 1020863 from the USDA National Institute of Food and Agriculture. This publication was also supported by the Nutrition and Obesity Policy Research and Evaluation Network (NOPREN). NOPREN is supported by Cooperative Agreement Number U48DP006374 funded by the Centers for Disease Control and Prevention’s (CDC) Division of Nutrition, Physical Activity, and Obesity and Prevention Research Centers Program. The findings and conclusions in this publication are those of the authors and do not necessarily represent the official position of USDA, the CDC, or DHHS. The funders were not involved in the preparation of this publication.
